# Generation of self-clusters of galectin-1 in the farnesyl-bound form

**DOI:** 10.1038/srep32999

**Published:** 2016-09-14

**Authors:** Kazumi Yamaguchi, Yusuke Niwa, Takakazu Nakabayashi, Hirotsugu Hiramatsu

**Affiliations:** 1 Graduate School of Pharmaceutical Sciences, Tohoku University, Sendai 980-8578, Japan

## Abstract

Ras protein is involved in a signal transduction cascade in cell growth, and cluster formation of H-Ras and human galectin-1 (Gal-1) complex is considered to be crucial to achieve its physiological roles. It is considered that the complex is formed through interactions between Gal-1 and the farnesyl group (farnesyl-dependent model), post-translationally modified to the C-terminal Cys, of H-Ras. We investigated the role of farnesyl-bound Gal-1 in the cluster formation by analyzing the structure and properties of Gal-1 bound to farnesyl thiosalicylic acid (FTS), a competitive inhibitor of the binding of H-Ras to Gal-1. Gal-1 exhibited self-cluster formation upon interaction with FTS, and small- and large-size clusters were formed depending on FTS concentration. The galactoside-binding pocket of Gal-1 in the FTS-bound form was found to play an important role in small-size cluster formation. Large-size clusters were likely formed by the interaction among the hydrophobic sites of Gal-1 in the FTS-bound form. The present results indicate that Gal-1 in the FTS-bound form has the ability to form self-clusters as well as intrinsic lectin activity. Relevance of the self-clustering of FTS-bound Gal-1 to the cluster formation of the H-Ras–Gal-1complex was discussed by taking account of the farnesyl-dependent model and another (Raf-dependent) model.

Ras proteins belong to a class of protein called GTPase and are involved in cell growth, differentiation, and cell death. Ras is distributed in the cytoplasmic side of the plasma membrane and plays an important role in various signal cascades, including cellular signal transduction^1^,^2^. Ras in complex with proteins other than Ras alone is considered to regulate physiological functions. The Ras family includes H-Ras, K-Ras, and N-Ras, and the amino acid sequences of these three proteins are 90% homologous with each other. Significant divergence among the Ras proteins appears in the C-terminal sequence, which is referred to as the hypervariable region (HVR). The C-terminal CAAX motif in the HVR of Ras proteins is post-translationally processed to generate an S-farnesylcysteine carboxymethyl ester; i.e., ^180^GCMSCKCVLS^189^ becomes ^180^GCMSCKC^186^-COOMe in the HVR of H-Ras^3^. The farnesyl group is reported to mediate protein–protein interactions^4^,^5^.

Human galectin-1 (Gal-1) is a member of the galectin family and has a specific affinity to β-galactosides. This globular protein comprises two β-sheet structures (β-sandwich structure) that create its carbohydrate recognition domain (CRD)^6^, and the amino acid sequence as well as the β-sandwich structure is highly conserved among the members of galectin family^7^,^8^. Despite the conserved structure of CRD and the sequence, specificity to glycans is different among the members of the galectin family^9^,^10^. Their role consequently differs with each other^11^. Physiological potential of Gal-1 has been known in regeneration of nerve cells^12^, angiogenetic effects^13^,^14^, apoptosis of T-cells^15^, and so on. Possible roles of Gal-1 are interesting in cancer biology because this protein is upregulated in cancer cells from bladder, thyroid, endometrial adenocarcinoma^16^,^17^. Gal-1 plays important roles in regulation of transformation, metastasis, and immune responses in tumor cells (see refs 18 and 19 for extensive review).

Gal-1 is linked to some physiological functions of the Ras proteins on the cell membrane, especially those related to cell signaling. It is considered that Gal-1 is a component of the H-Ras cluster^20^ and a receptor of the farnesyl group of H-Ras^21^, i.e., Gal-1 and H-Ras exhibit the complex through the interaction between Gal-1 and the farnesyl chain of H-Ras (the farnesyl-dependent model)^22^. Involvement of the farnesyl group in the formation of clusters is also supported by the fact that farnesyl thiosalicylic acid (FTS), a small molecule having the farnesyl group, inhibits the Ras-dependent cell growth^23^,^24^. The formation of the clusters of the H-Ras–Gal-1 complex results in the activation of physiological functions, such as the Raf/MEK/ERK pathway^25^, and the downregulation of Gal-1 expression decreases the number of H-Ras(G12V) clusters at the plasma membrane^26^. Furthermore, FTS dislodges the Ras clusters from cell membranes^27^ and has therapeutic potential for pancreatic cancer (under the product name Salirasib)^28^,^29^. The site of Gal-1 to which the farnesyl chain binds is still unclear, but the farnesyl group of H-Ras is considered to be inserted between the two β-sheets of Gal-1^22^,^30^.

Information on the structure and properties of Gal-1 in the farnesyl-bound form is important to elucidate the cluster formation mechanism of H-Ras with Gal-1. In the present study, we investigated the role of Gal-1 in the H-Ras–Gal-1 complex by analyzing the structure and properties of Gal-1 in the FTS-bound form. We have shown in this study that Gal-1 in the farnesyl-bound form acquires the ability to form self-clusters, and the galactoside-binding pocket of Gal-1 in the FTS-bound form plays an important role in self-cluster formation. Effects of the self-clustering of FTS-bound Gal-1 on the formation of the clusters of the complex of H-Ras and Gal-1 was discussed by taking into account the farnesyl-dependent model and Raf-dependent model that is recently proposed.

## Results

### FTS induces Gal-1 self-clustering

**Figure 1:**
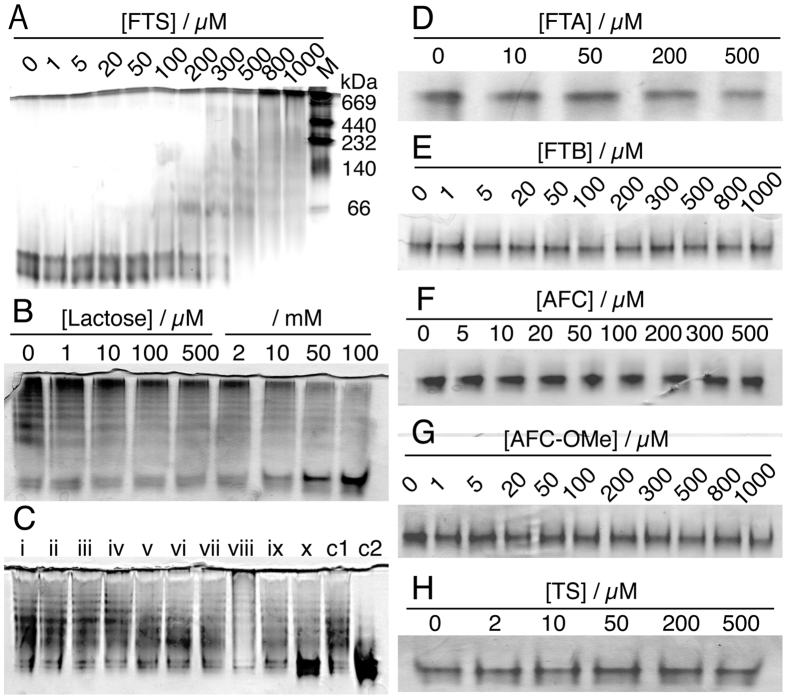
Images of native gel electrophoresis of Gal-1 with different concentrations of FTS (**A**) and in the presence of 500 μM FTS with (**B**) different lactose concentrations, (**C**) with saccharides [glucose (i; 1 mM, ii; 100 mM), L-galactose (iii; 1 mM, iv; 100 mM), D-galactose (v; 1 mM, vi; 100 mM), D-galactosamine (vii; 1 mM, viii; 100 mM), lactose (ix; 1 mM, x; 100 mM), no reagents (c1), or Gal-1 only (without FTS) (c2)], or with different concentrations of FTA (**D**), FTB (**E**), AFC (**F**), AFC-OMe (**G**), and TS (**H**). M in (**A**) denotes the lane of marker bands of the molecular weight.

We report Gal-1 self-cluster formation via an interaction with FTS. Figure 1A shows the images of native polyacrylamide gel electrophoresis of Gal-1 with increasing concentration of added FTS. A single band is observed when the FTS concentration is <100 μM. The observed single band corresponds to the Gal-1 dimer (29.4 kDa). Multiple bands having low mobility appear in the range of 100–1000 μM, indicating efficient Gal-1 cluster formation with FTS addition. Comparing with the marker bands, the self-cluster as large as ca. 232 kDa is detected in the presence of 500 μM FTS. The molecular weight of this large cluster is comparable to that of eight Gal-1 dimers (235 kDa). Multiple bands at 500 μM FTS are markedly reduced when lactose, which binds to the galactoside-binding pocket in Gal-1, is added to the Gal-1 solution (Fig. 1B); however, the same effect was not observed when saccharides having low affinity to Gal-1^31^ are added (Fig. 1C). Furthermore, multiple bands are not induced with the addition of other farnesyl derivatives, such as farnesyl thioacetic acid (FTA) (Fig. 1D), farnesyl thiobenzene (FTB) (Fig. 1E), *N*-acetyl-S-farnesyl-L-cysteine (AFC) (Fig. 1F), *N*-acetyl-S-farnesyl-L-cysteine methyl ester (AFC-OMe) (Fig. 1G), or thiosalicylic acid (TS) (Fig. 1H), indicating that Gal-1 clustering does not occur with other farnesyl derivatives at the present range of concentrations. Gal-1 is found to gather also at high concentration without FTS (see Supplementary Fig. S1A), while this gathered form can be regarded to be different from the self-clusters with FTS because the gathered form does not show the ladder-like pattern in the gel image.

### FTS makes Trp68 environment polar

**Figure 2:**
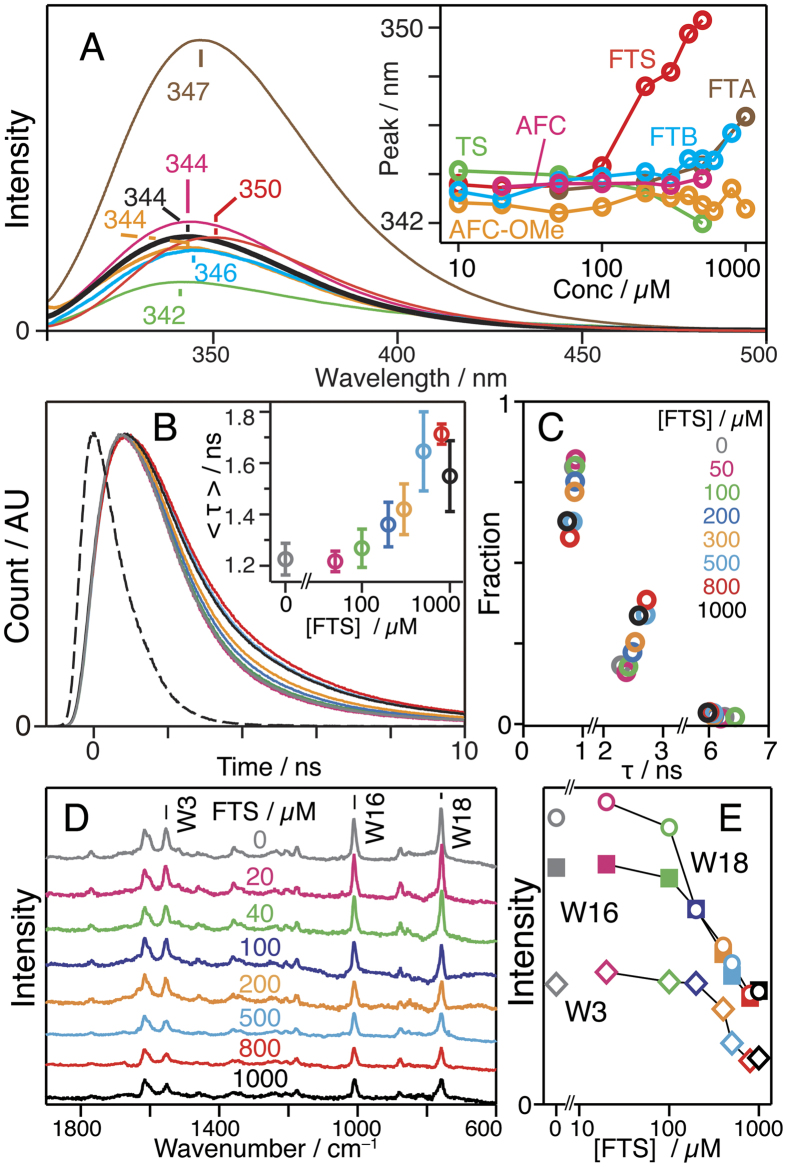
Spectroscopic results for the analysis of Trp68 environment. (**A**) Fluorescence spectra of Gal-1 (black), Gal-1 with 500 μM FTS (red), 1500 μM FTA (brown), 800 μM FTB (blue), 500 μM AFC (magenta), 1000 μM AFC-OMe (orange), and 500 μM TS (green). (**A**, inset) Fluorescence peak position plotted against the concentration of each compound. (**B**) Fluorescence decay curves at different FTS concentrations. Instrumental response function is shown by a chain line. (**B**, inset) Average fluorescence lifetime of Trp68 plotted against FTS concentration. (**C**) Fraction of each component of the fluorescence decay plotted against its lifetime. (**D**) The 229-nm excited UV resonance Raman spectra at different FTS concentrations. (**E**) Band intensities of W3 (◊), W16 (■), and W18 (○) plotted against FTS concentration.

The addition of farnesyl derivatives changes the fluorescence spectrum of Trp68, which is the unique Trp in the subunit of Gal-1 and is one of the residues constituting the galactoside-binding pocket (Fig. 2A). The addition of FTS, FTA, or FTB induces a red shift in the fluorescence, suggesting an increase in the polarity (hydrophilicity) of the environment around Trp68^32^, whereas the peak of the fluorescence remains unchanged with the addition of AFC, AFC-OMe, or TS. FTS induces the largest shift in fluorescence among the farnesyl derivatives used in the present study (Fig. 2A, inset). The fluorescence decay profile of Trp68 also changes with increasing concentrations of FTS (Fig. 2B). The average fluorescence lifetime <τ>, which is evaluated by assuming a tri-exponential decay (*i* = 1–3; see Materials and Methods), remains constant at ~1.2 ns in the 0–100 μM range, increases to ~1.7 ns in the 100–800 μM range, and is slightly reduced in the 800–1000 μM range (Fig. 2B, inset). Figure 2C shows the fractions of the *i*-th component plotted against τ_*i*_. The increase in <τ> is mainly due to the decrease in the fraction of the first component (τ_1_ = ~1 ns) and the concomitant increase in the second component (τ_2_ = 2–3 ns). The contribution of the third component (τ_3_ = ~6 ns) is negligible.

Figure 2D shows the UV resonance Raman spectra of Gal-1 with different concentrations of FTS. The Raman bands of Trp68 (W3, W16, and W18) are clearly observed, which is due to the resonance effect of the B_b_ absorption band of Trp with the excitation wavelength of 229 nm. The intensity of these bands is reduced with increasing concentrations of FTS from 100 μM to 800 μM, and remains unchanged in the 800–1000 μM range (Fig. 2E). The decrease in the Raman intensity of Trp comes from the reduction of the resonance effect with the B_b_ absorption band, which is attributed to the increase in the polarity of the environment of Trp^33^. This result is consistent with the fluorescence results mentioned above, leading us to conclude that the environment of Trp68 in Gal-1 becomes polar with increasing FTS concentrations. This result is in contrast to the case of lactose binding, which makes the environment of Trp68 more hydrophobic (see Supplementary Fig. S2)^34^. Hence, the change in the Trp68 environment with FTS has several steps: the environment remains unchanged in the 0–100 μM range, the polarity increases in the 100–800 μM range, and the environment becomes constant in the 800–1000 μM range. We also observed the similar changes in the circular dichroism (CD) spectrum of Gal-1 with increasing FTS concentration (see Supplementary Fig. S3), indicating that the conformational change of Gal-1 occurs in conjunction with the change in the environment of Trp68.

**Figure 3:**
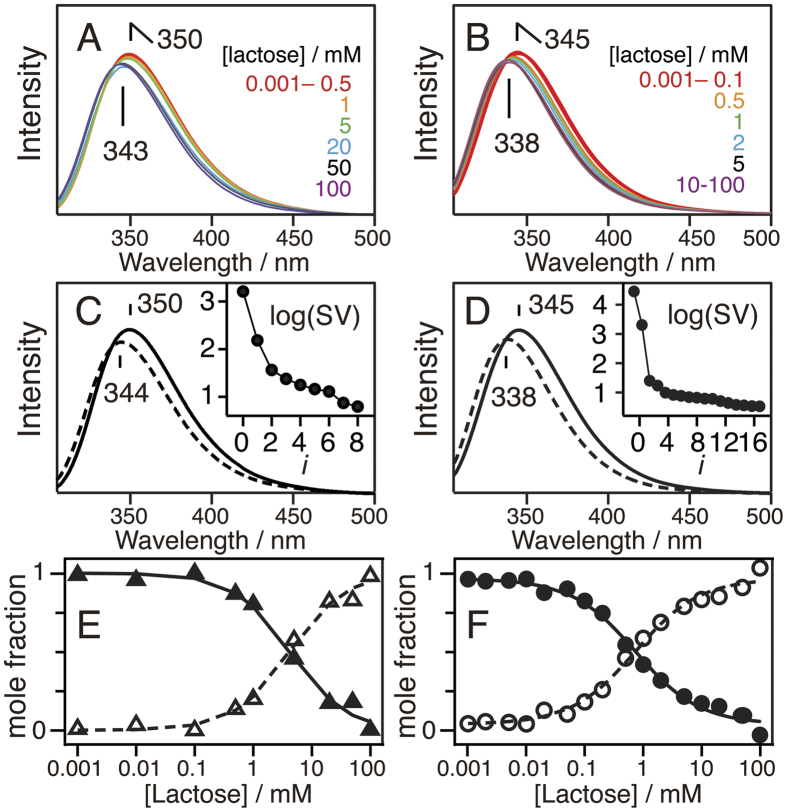
(**A**,**B**) Fluorescence spectra of 5.1 μM Gal-1 at pH 7.4 and 25% (v/v) EtOH in the presence of different lactose concentrations (0.001–100 mM). The fluorescence was excited at 295 nm. (**C**,**D**) Calculated fluorescence spectra for free (—) and lactose-bound (---) forms. The spectra were extracted from the experimental spectra by the SVD-based equilibrium analysis, and a singular value plot is shown in the inset. (**E**,**F**) Mole fractions of the free (solid symbols) and lactose-bound (open symbols) forms of Gal-1 are concomitantly obtained. Mole fractions calculated from the equilibrium between lactose-free and lactose-bound states (Equation (3)) are shown by solid lines. The calculations were performed using the values of *K*_b_ and *n* in the text. Left panels (**A**,**C**,**E**) are the results in the presence of 500 μM FTS, and right panels (**B**,**D**,**F**) are those in the absence of FTS.

It is expected that the lectin activity of Gal-1 is affected by the interaction with FTS because the environment of Trp68 constituting the galactoside-binding pocket changes with the addition of FTS, and the amount of the clusters is reduced in the presence of lactose. We therefore evaluated the lactose-binding constant *K*_b_ of Gal-1 in the presence and absence of 500 μM FTS. As shown in Fig. 3, the fluorescence of Trp68 shifts to a shorter wavelength with increasing lactose concentration irrespective of the existence of FTS, and the significant blue shift in the peak position occurs with a lactose concentration of 0.5–20 mM in the presence of 500 μM FTS (Fig. 3A) and that of 0.1–10 mM in the absence of FTS (Fig. 3B). This indicates that the presence of FTS lowers the lactose-binding affinity of Gal-1.

### Affinity to Lactose lowers in FTS-bound form of Gal-1

The dependence of the fluorescence spectra on lactose concentration was quantitatively analyzed using singular value decomposition (SVD) analysis^35^. The two values are effectively large in the singular value plot irrespective of the existence of FTS (inset in Fig. 3C,D), suggesting that the binding of lactose is described by the equilibrium between the lactose-free and lactose-bound forms of Gal-1. The obtained change in the molar fraction of the lactose-free and lactose-bound forms with increasing concentration of FTS was fitted with the two-state equilibrium model (Equation (3), see Materials and methods). The present *K*_b_ and Hill coefficient *n* are 0.58 ± 0.14 mM^−1^ and 0.91 ± 0.16, respectively, in the presence of 500 μM FTS, and 1.36 ± 0.17 mM^−1^ and 0.80 ± 0.20, respectively, in the absence of FTS. The *K*_b_ value was confirmed to be lowered in the presence of 500 μM FTS, leading us to conclude that the Gal-1 clusters with FTS have a lower lactose-binding affinity than that of isolated Gal-1. It should be noted that the peak position of the fluorescence spectrum of the lactose-bound form shifts to a longer wavelength with the addition of FTS (Fig. 3C,D). This result indicates that Gal-1 simultaneously binds both lactose and FTS, meaning that the binding site of FTS is different from that of lactose in Gal-1.

### FTS binding changes structure of lactose-binding pocket of Gal-1

**Figure 4:**
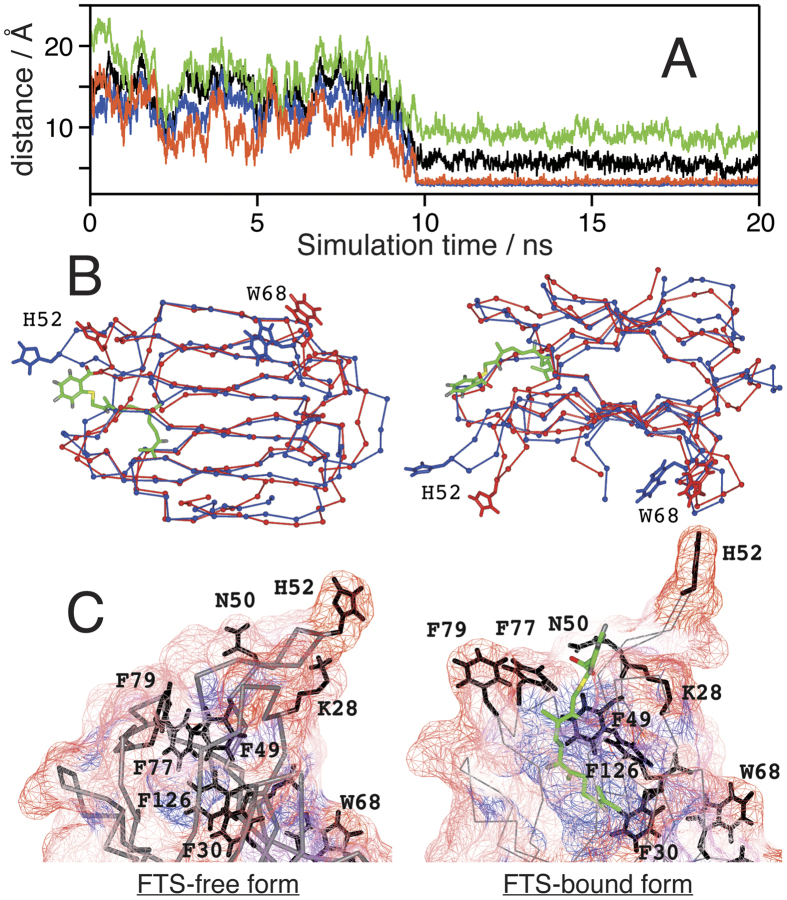
MD simulations of Gal-1 and its FTS-bound form. (**A**) Distance between residues of Gal-1 and/or functional groups of FTS. Orange, Lys28 (side chain N)-FTS (C5 of the benzene ring); blue, Asn50 (main chain N)-FTS (O1 of the carboxylate); black, Asn50 (side chain N)-FTS (O2 of the carboxylate); green, His52 (Cα)-FTS (C5 of the benzene ring). (**B**) Superimposed structures of free Gal-1 (red) and the FTS-bound form (blue) at 20 ns. The FTS molecule is illustrated with green. (**C**) Structures around the FTS binding site of the FTS-free (left) and FTS-bound (right) forms. Mesh surface shows the solvent accessibility of each residue: red (high)-white-blue (low).

Finally, we performed a molecular dynamics (MD) simulation of the interaction between the Gal-1 monomer and FTS in the 20-ns range. Figure 4A shows the time course of the distance between some residues of Gal-1 and FTS. The benzene ring of FTS is calculated to be in the proximity of Lys28, Asn50, and His52 after ~10 ns. The representative structures with and without FTS at 20 ns are superimposed in panel B, and separately illustrated in panel C of Fig. 4, respectively. The binding site of FTS is different from that of lactose, which is in agreement with the experimental results mentioned above and the MD simulation previously reported^22^. The binding of FTS induces the change in the structure of a loop at Asn50-Ala55 (Fig. 4B,C). From the calculated geometry, the binding interaction between Gal-1 and FTS is as follows: the hydrogen bond between the COO^−^ group of FTS and the amide protons of Asn50, the cation–π interaction^36^,^37^ between the aromatic ring of FTS and Lys28, and the hydrophobic interaction between the farnesyl group and the phenylalanine side chains (Phe30, Phe49, Phe126). It should also be noted that the hydrophilic residues of Phe77 and Phe79, which are buried in Gal-1, move to the protein surface and become exposed to the solvent after the binding to FTS (Fig. 4C).

## Discussion

We showed that Gal-1 forms self-clusters with binding to FTS (Fig. 1(a)), and the environment of Trp68 that constitutes the galactoside-binding pocket of Gal-1 changes with self-cluster formation (Fig. 2). The environment around Trp68 becomes polar with increasing FTS concentrations of 100–800 μM and then remains unchanged at high FTS concentrations of 800–1000 μM. The change in the environment of Trp68 occurs in conjunction with the cluster-formation process in which small-size clusters are formed in the 100–800 μM range, and only large-size clusters with very low mobility appear in the 800–1000 μM range. It is therefore concluded that small-size cluster formation increases polarity around Trp68, and the environment of Trp68 remains unchanged with large-size cluster formation. In the measurements with the various farnesyl derivatives, not only Gal-1 clustering (Fig. 1) but also marked shift in fluorescence (Fig. 2A) occurs only when FTS is added to the solution. The present observation does not come from non-specific hydrophobic interactions between Gal-1 and the farnesyl derivative and specific interactions between Gal-1 and FTS are necessary to generate Gal-1 clusters. The MD simulation suggests that the possible interaction of Gal-1 and FTS is between the COO^−^ of FTS and Asn50, between the benzene ring of FTS and Lys28, and between the farnesyl group and the phenylalanine side chains (Fig. 4C). The very low affinity of Gal-1 to FTA (Fig. 1D), to FTB (Fig. 1E), and to TS (Fig. 1H) corresponds with this calculation, as they do not have the COO^−^ group, the benzene ring, or the farnesyl group, respectively.

The galactoside-binding pocket of Gal-1 can be considered to play an important role in small-size cluster formation because the environment of Trp68 constituting the galactoside-binding pocket is affected by the addition of FTS (Fig. 2), and the existence of lactose decreases the amount of the small-size clusters (Fig. 1B). This conclusion is confirmed by the fact that small-size cluster formation lowers the lactose-binding affinity of Gal-1 (Fig. 3). Lactose is not readily accessible to the binding pocket of Gal-1 in the small-size clusters, suggesting that the small-size clusters with FTS are generated with the interaction among the carbohydrate-binding pockets of Gal-1. The interaction leading to the cluster formation may be prompted with the deformation of the Asn50–Ala55 loop (Fig. 4B), because the native structure of soluble protein is designed to avoid the self-clustering^38^ (Fig. 1A). It should be noted that the midpoint of the change in the Trp68 environment is ca. 300 μM (Fig. 2), while the half maximum of effective concentration of FTS to inhibit growth of Ras-transformed cells (EC_50_, 7.5 ± 3.7 μM)^23^. This difference may be due to the fact that the clustering of Ras proteins occurs on the membrane with very dense concentration, which is different from the present experimental condition of an isolated state in aqueous solution. The FTS concentration dependence of the results in Fig. 2 is analyzed by assuming the equilibrium between the ligand-free and ligated structures (Equation (3)). The dissociation constant (inverse of *K*_b_) and the Hill coefficient *n* of the FTS binding are derived to be ca. 2.3 μM and 11, respectively. The large value of *n* implicates that the FTS binding of Gal-1 is cooperative.

The increase in the polarity around Trp68 with small-size cluster formation may be attributed to the conformational change of Gal-1 caused by the binding of FTS, which is clarified by the CD spectra with different FTS concentrations (see Supplementary Fig. S3). One of the possible origins of the change in the Trp68 environment is the deformation of the Asn50–Ala55 loop in the carbohydrate-binding pocket (Fig. 4B). The observed peak shift from 343 nm to 350 nm (Fig. 1A, inset) corresponds to a change in the local electric field as large as 9 MV cm^−1^ along the direction from the benzene to the pyrrole ring^39^. Such a large change in the local electric field would not be attributed solely to rearrangement of the side chains. It is conceivable that Trp68 is moved from the hydrophobic environment embedded in the protein to the hydrophilic environment partly exposed to the aqueous solvent.

Furthermore, the change in <τ> with small-size cluster formation is explained in terms of the change in the population of Trp68 between the hydrophobic and hydrophilic environments. As shown in Fig. 2C, the two lifetime components (τ_1_ and τ_2_) are dominant in the fluorescence decay of Trp68 irrespective of FTS concentration, and the contribution of the τ_2_ component increases with increasing concentration of FTS, resulting in the increase in <τ> in the FTS concentration of 100–800 μM. The τ_1_ and τ_2_ components can be assigned to Trp68 in the hydrophobic and hydrophilic environments, respectively, which comes from the fact that the value of τ_1_ is almost independent of the FTS concentration and that of τ_2_ has a tendency to increase with FTS concentration. Trp68 in the hydrophobic environment is embedded in the protein, resulting in the effective protection from water molecules^6^. Therefore, the fluorescence lifetime of Trp68 in the hydrophobic environment is expected to be almost independent of the FTS concentration. On the other hand, Trp68 in the hydrophilic environment is exposed to water molecules, the magnitude of which depends on the FTS concentration. The fluorescence lifetime of Trp68 in the hydrophilic environment is therefore affected by the FTS concentration.

The generation of the large-size clusters at high FTS concentration of 800–1000 μM is not accompanied by a change in the polar environment of Trp68, indicating that the interaction site among Gal-1 forming the large-size clusters is different from that of the small-size clusters. The galactoside-binding pocket seems to not contribute to the generation of the large-size clusters. A possible interaction among Gal-1 in the large-size clusters is the hydrophobic interaction using Phe77 and Phe79 at the farnesyl-binding site, which become exposed to the solvent in the FTS-bound form (Fig. 4C).

According to the proposed farnesyl-dependent model, the formation of the H-Ras clusters is explained in terms of the clustering of Gal-1 binding the farnesyl chain of H-Ras^22^. The present result is consistent with this model that the formation of Gal-1 self-clusters in the farnesyl-bound form is responsible for H-Ras cluster formation *in vivo*. However, the affinity of AFC-OMe, a model compound of the C-terminal Cys of H-Ras, to Gal-1 is lower than that of FTS (Figs 1G and 2A) because either the phenyl ring or the carboxyl group, which is considered to be important for FTS to bind to Gal-1, is absent in AFC-OMe. This result also suggests the necessity of the reconsideration of the farnesyl-dependent model. Possible explanation of our result is that FTS is an efficient competitive inhibitor for the binding of H-Ras to Gal-1, and this is why FTS efficiently degrades the Ras clusters from the cell membrane^27^.

Blaževitš *et al*. have recently proposed a new model in which H-Ras recruits Raf effectors and forms the H-Ras–Raf complexes. The complexes are bridged with the Gal-1 dimer attached to the Ras binding domain in Raf, resulting in the cluster formation (Raf-dependent model)^40^. They concluded that the formation of the clusters did not require the direct interaction between the farnesyl group and Gal-1, because neither the K28T mutant of Gal-1, which is reported to have no affinity to the farnesyl group, nor the C186S mutant of H-RasG12V (the farnesyl group is abolished) showed any decrease in the FRET efficiency from GFP of H-RasG12V to RFP of Gal-1^40^. According to the Raf-dependent model, the effect of the addition of FTS on the Ras-dependent cell growth^23^,^24^ is explained as follows: the formation of the self-clusters of Gal-1 with FTS decreases both the number of Gal-1 bridging the H-Ras–Raf complexes and that of the clusters of H-Ras–Raf–Gal-1 complex on the membrane. The present low affinity of AFC-OMe to Gal-1 (Figs 1G and 2A) supports this Raf-dependent model. It should be noted, however, that both the wild-type (Fig. 1A) and the K28T mutant (see Supplementary Fig. S1B) of Gal-1 exhibited the self-clusters in the presence of 200 μM FTS. Therefore, local concentration of the farnesyl group on the membrane may be a key factor to clarify the detailed mechanism of the cluster formation of H-Ras.

On the membrane, Gal-1 is assembled as the dimer in the cluster with H-Ras in both the farnesyl-dependent^22^ and the Raf-dependent^40^ models. The local concentration of Gal-1 on the membrane is probably higher than that in serum of healthy individuals (<13.5 ng mL^−1^)^41^. Properties of the Gal-1 dimer rather than the monomer should be responsible for the cluster formation of Gal-1, and the dimeric form of Gal-1 was investigated in our study for this reason. Gal-1 exists in a monomer-noncovalent dimer equilibrium, and the fraction of monomer increases below 2 μM^42^.

Finally, the farnesyl-binding site has been used to suppress abnormal Ras signaling, and FTS succeeded in reducing the cluster of H-Ras and Gal-1^27^. The H-Ras–Gal-1 clusters *in vivo* seem to be the small-size clusters in the present study, judging from the number of protein subunits^43^. The present study indicates that lactose also interferes with cluster generation (Fig. 1B). β-galactoside may also be used as an inhibitor to decrease the clusters.

## Methods

### Sample preparation

The procedures for protein expression and purification have been described elsewhere^35^. Briefly, Gal-1 was dissolved into double-distilled water and was incubated in aqueous 8 mM β-mercaptoethanol for 3 h at pH 8.2, 37 °C to remove the oxidized form that is inactive in galactoside binding^12^. The solution was then mixed with the same volume of a solution containing 40 mM phosphate, 40 mM NaCl, 50% (v/v) EtOH, and the farnesyl derivatives and/or the saccharides. EtOH was used to solubilize the farnesyl derivatives. Gal-1 concentration was 10 μM (0.15 mg ml^−1^) with the fluorescence measurement and 68 μM (1.0 mg/mL) with the Raman measurement. Gal-1 concentration was determined in monomer unit using the molar extinction coefficient (8,480 M^−1 ^cm^−1^ at 280 nm) calculated from the numbers of Trp and Tyr residues in the protein^44^. In the Raman measurement, 2 mM NaNO_3_ was added to the sample solution, and the 1047 cm^−1^ Raman band of NO_3_^−^ was used as an internal intensity standard. Lactose (Nacalai Tesque, Kyoto, Japan), TS (Sigma-Aldrich, St. Louis, MO), FTA (Enzo life Sciences, Farmingdale, NY), AFC (Cayman, Ann Arbor, MI), and AFC-OMe (BACHEM, Bubendorf, Switzerland) were used without further purification. FTB was synthesized using thiobenzene as an initial compound (see Supplementary information)^23^.

### Acquisition of spectra

Fluorescence spectra were recorded on a spectrofluorometer (FP-6500, JASCO) with the excitation wavelength of 295 nm. The fluorescence decay profile was measured using the time-correlated single photon counting method (FluoroCube UltraFast-3000U, Horiba). The excitation and detection wavelengths were 279 nm and 343 nm, respectively. The fluorescence decay curve *f*(*t*) was fitted with a tri-exponential function (Equation (1)) convoluted with the response function.


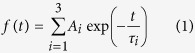


where *A*_*i*_ and τ_*i*_ are the pre-exponential factor and fluorescence lifetime of the *i*-th component, respectively. The average lifetime and the fraction of the *i*-th component were evaluated as follows.


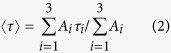


UV Raman spectra were measured with the excitation wavelength of 229 nm using a frequency-doubled Ar^+^ laser (Innova 300 FReD, Coherent). The scattered Raman signal was detected with a liquid-nitrogen-cooled CCD detector (LN/CCD-1152, Princeton Instruments). Raman signals were accumulated for 20 s per spectrum, and 75 spectra were averaged.

### Native polyacrylamide gel electrophoresis

Separation gel (15 wt% acrylamide) and concentration gel (3 wt% acrylamide) were prepared. An aliquot of Gal-1 solution was applied to each lane with the tested compound(s), e.g., FTS, lactose, etc., and the electric field was applied for 85 min using Pt electrodes with the constant current mode (20 A). The buffer was composed of 25% (v/v) EtOH, 4 mM β-mercaptoethanol, and 25 mM Tris-Glycine (pH 8.3). The concentration of Gal-1 was set to 10 μM. Molecular weight marker (HMW Native Marker Kit, GE Healthcare, IL) was also applied for the reference. The obtained bands were visualized with silver staining (Fig. 1A–C) or with Coomassie Brilliant Blue dye (Fig. 1D–H).

Equilibrium Analyses of Ligand Binding. The binding of ligand (L) to protein (P) was analyzed by assuming the equilibrium between ligand-free and ligand-bound states:


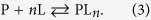


The apparent binding constant *K*_B_ and the Hill coefficient *n* were obtained from the SVD analysis of the spectroscopic data. The obtained *K*_B_ is equal to (*K*_b_)^*n*^, where *K*_b_ is the binding constant of ligand at each step^45^.

## Additional Information

**How to cite this article**: Yamaguchi, K. *et al*. Generation of self-clusters of galectin-1 in the farnesyl-bound form. *Sci. Rep.* **6**, 32999; doi: 10.1038/srep32999 (2016).

## Supplementary Material

Supplementary Information
