# Supplementary material for: Generation of self-clusters of galectin-1 in the farnesyl-bound form
Source: Sci Rep. 2016 Sep 14;6:32999. doi: 10.1038/srep32999 (PMC5021961; doi:10.1038/srep32999)
Supplement: Supplementary Information [file srep32999-s1.doc]

SUPPLEMENTARY INFORMATION

**Generation of self-clusters of galectin-1 in the farnesyl-bound form**

Kazumi Yamaguchi, Yusuke Niwa, Takakazu Nakabayashi, and Hirotsugu Hiramatsu*

Graduate School of Pharmaceutical Sciences, Tohoku University, Sendai 980-8578, Japan.

* Correspondence should be addressed to H.H. (hiramatu@m.tohoku.ac.jp)

**Synthesis of farnesyl thiobenzene (FTB).** Farnesyl thiobenzene (FTB) was prepared on the basis of the synthesis of farnesyl thiosalicylic acid (FTS):^1^ thiophenol instead of thiosalicylic acid was adopted as an initial compound in the reaction.

Thiophenol (0.2 g, 2 mmol), guanidine carbonate (0.4 g, 2.3 mmol), and trans,trans-famesyl bromide (0.6 g, 2 mmol) were mixed overnight in 25 mL of acetone at room temperature. After filterating guanidine carbonate and evapolating acetone, chloroform was added together with a few drops of 2 N HC1. The mixture was washed with water for three times, and the organic phase was separated, dried on magnesium sulfate and then evaporated. Yellowish oil was obtained. The product was purified on silica gel with mixtures of chloroform and ethyl acetate (5:1–1:5) and then ethyl acetate as eluents. ^1^H-NMR (JNM-ECA700, JEOL, Japan) (CDCl_3_) δ1.58 (12H, m), 2.02 (8H, m), 3.54 (2H, d), 5.08 (2H, q), 5.30 (1H, t), 7.17 (1H, t), 7.26 (2H, t), 7.33 (2H, d).

**Molecular Dynamics Simulation.** Primergy RX300 S7 (Fujitsu, Japan) with GROMACS (Groningen Machine for Chemical Simulation) software^2^ in Research Center for Computational Science (Japan) was used for the calculation. GROMOS 53A6 force field parameters of FTS was obtained from Automated Topology Builder.^3,4^ The obtained structures were illustrated using CCP4mg.^5^ The simulations were carried out for the Gal-1 monomer species. As a solution structure of Gal-1, the atomic coordinate in the first model of PDB file 2KM2 was used, in which His44 and His52 existed as the N_π_-H and N_τ_-H forms, respectively.^6^ The molecule(s) was placed in the center of a periodic truncated cubic box, which was subsequently filled with simple point-charge water molecules. The distances between the atoms in the protein and the wall of the periodic box were at least 0.7 nm, and sodium ions were added to make the total charge of the box zero.

Prior to the MD simulation, the potential energy was minimized using a steepest-descent algorithm, followed by a 20-ps MD simulation with position restraints of the atoms in the protein to equilibrate the location of the added solvent molecules. Then the system was repeatedly heated and equilibrated for 2 ns, and a structure having the lowest potential energy in this trajectory was chosen as the initial structure of the MD simulation for 20 ns. The result of the 20-ns simulation was analyzed. The MD simulation was carried out for the ensemble of constant pressure and temperature with periodic boundary conditions. The temperature and pressure were maintained at 310.15 K and 1 bar by coupling to an external heat and an isotropic pressure bath. The compressibility was set to 4.5 × 10^5^ bar in all box dimensions. Coulomb interactions were treated using the fast particle-mesh Ewald (PME) method, and non-bonded interactions were generated using a twin-range method.^7^ The cutoffs of both interactions were 1.4 nm and were updated every 10 steps. The LINCS algorithm was used to constrain bond lengths.^8^ The simulation time-step was 2 fs and coordinates were saved for analysis of the results in the every 1 ps.

**Examination of self-clustering.** Experimental conditions of native polyacrylamide gel electrophoresis are identical to those described in the main text. Molecular weight marker (HMW Native Marker Kit, GE Healthcare, IL) was also applied in the lanes indicated with “M” for the reference. The obtained bands were visualized with [Coomassie Brilliant Blue](http://lsd-project.jp/weblsd/c/begin/Coomassie Brilliant Blue) dye (A) or with silver staining (B).

**A. Gal-1 concentration dependence.**

By changing the concentration of Gal-1, the size of self-clusters was analyzed in the presence or absence of FTS. Numbers on each lane indicate the concentration of FTS (upper) and Gal-1 (lower) in µM unit. Besides the self-clustering of the FTS-bound form, Gal-1 is found to gather at high concentration without FTS (Figure S1A). This gathered form can be different from the self-clusters formed in the presence of FTS, because the gathered form does not show the ladder-like pattern in the gel image.

**B. K28T mutant.**

The K28T mutant of Gal-1 is reported to abolish the property of farnesyl recognition.^9^ We tested whether or not the K28T mutant would lose the self-clustering propensity in order to verify our idea that the FTS binding was an origin of the self-clustering. The gel image in Figure S1B shows that the K28T mutant actually formed the multiple self-clusters in the presence of FTS, and the result is not largely different from that of the wild type Gal-1 (Figure 1A).

The ability of K28T to interact with the farnesyl group was assessed using farnesol, and reduced affinity was observed.^9^ However, the lowered affinity to farnesyl does not necessarily mean the reduction of the affinity to all of the farnesyl analogues because the affinity of Gal-1 to the farnesyl analogues is different from each other as we mentioned in Figure 1. Thus, the result in Figure S1B suggests that K28T remains the affinity to FTS and the affinity is likely comparable with that of the wild type Gal-1.

**UV resonance Raman spectra of Gal-1 in the presence of FTS and/or Lactose.** UV resonance Raman spectra were measured with 229-nm excitation. The concentration of Gal-1 was set to 68 µM (1.0 mg/mL). Several Raman bands of Trp68 of Gal-1 are observed (Figure S3, top): W3 at 1553 cm^–1^, W16 at 1010 cm^–1^, and W18 at 760 cm^–1^. The addition of 100 mM lactose increases the intensity of these bands, whereas the addition of 500 µM FTS decreases, which reflects the change in the polar environment of Trp; the increase/decrease of the Trp bands means the decrease/increase of the polar environment.^10^ The addition of lactose induces the apolar environment of Trp68, which is consistent with the previous report of Di Lella *et al.*^11^ It should be noted that in the presence of lactose and FTS, the intensity of the Trp bands is between those with lactose and FTS.

**FTS concentration dependence of CD spectrum of Gal-1.** CD spectra of Gal-1 were measured to investigate the change in the secondary structures due to the cluster formation (Fig. S3). The measurement was carried out by a spectropolarimeter (JASCO J-820) with a 1-mm cell and 2-nm spectral slit width. The CD spectrum in the absence of FTS shows the negative peak at around 216 nm, indicating the existence of the β-barrel structure.^12^ The spectrum remains unchanged with increasing concentration of FTS until 100 µM, and then the two-step change is observed in the range of 100 – 1000 µM; the magnitude of the peak decreases until 400 µM, and then increases with increasing FTS concentration (500–1000 µM). This result indicates that at least two kinds of the structural change occur in Gal-1 with increasing FTS concentration. The FTS concentration dependence was not observed in the CD spectrum when the other farnesyl derivatives (FTA, FTB, AFC, AFC-OMe, and TS) were added.


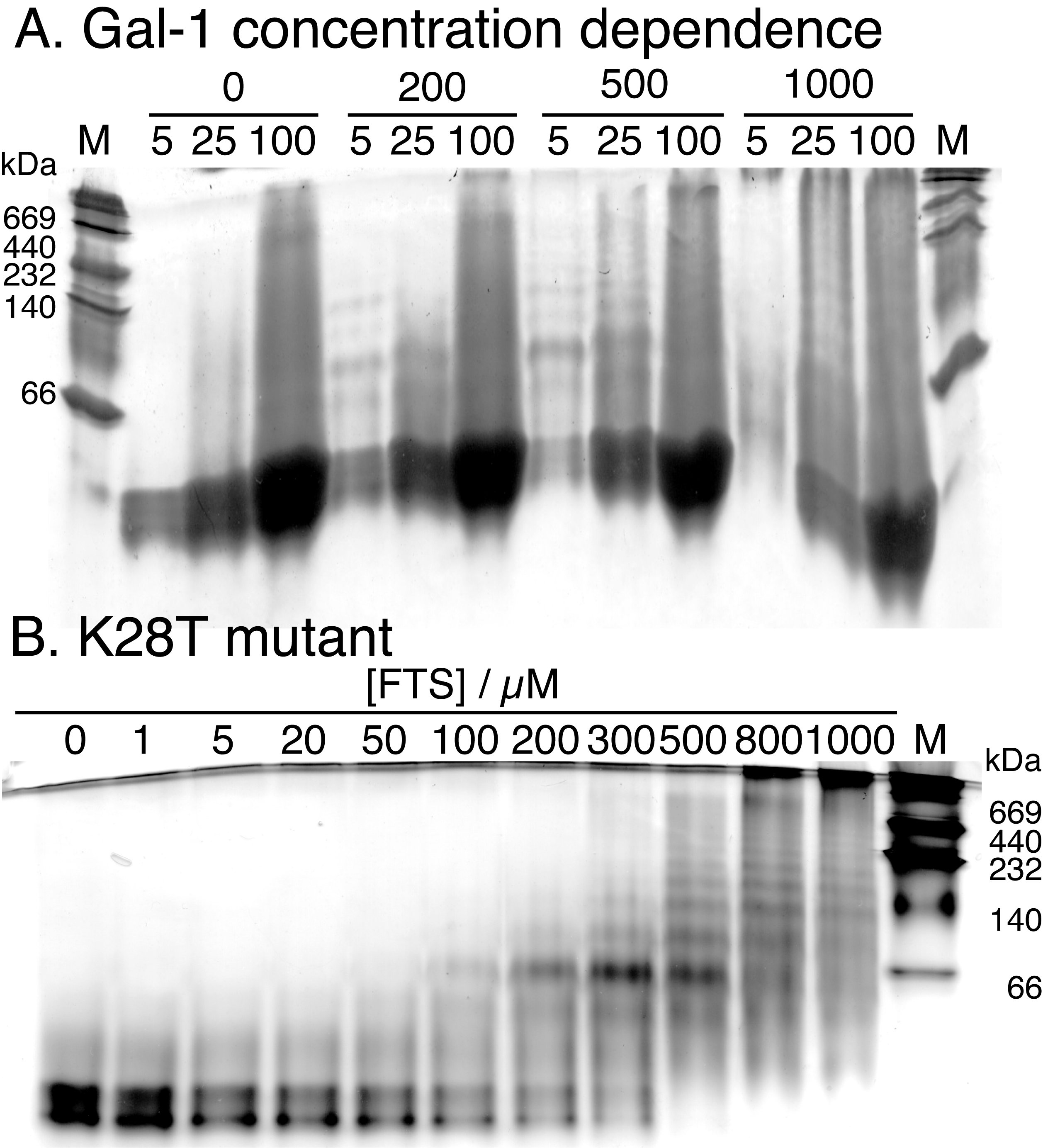


**Figure S1** Gel image of Native PAGE. A: Gal-1 concentration dependence with fixed concentration of FTS. The concentration of FTS and Gal-1 are shown in upper/lower columns over each lane. The molecular weight of marker bands (given in kDa) is shown by the side of lane M. B: FTS concentration dependence of K28T mutant of Gal-1. Concentration of FTS is shown on the top of each lane. The concentration of the protein is set to 10 µM.


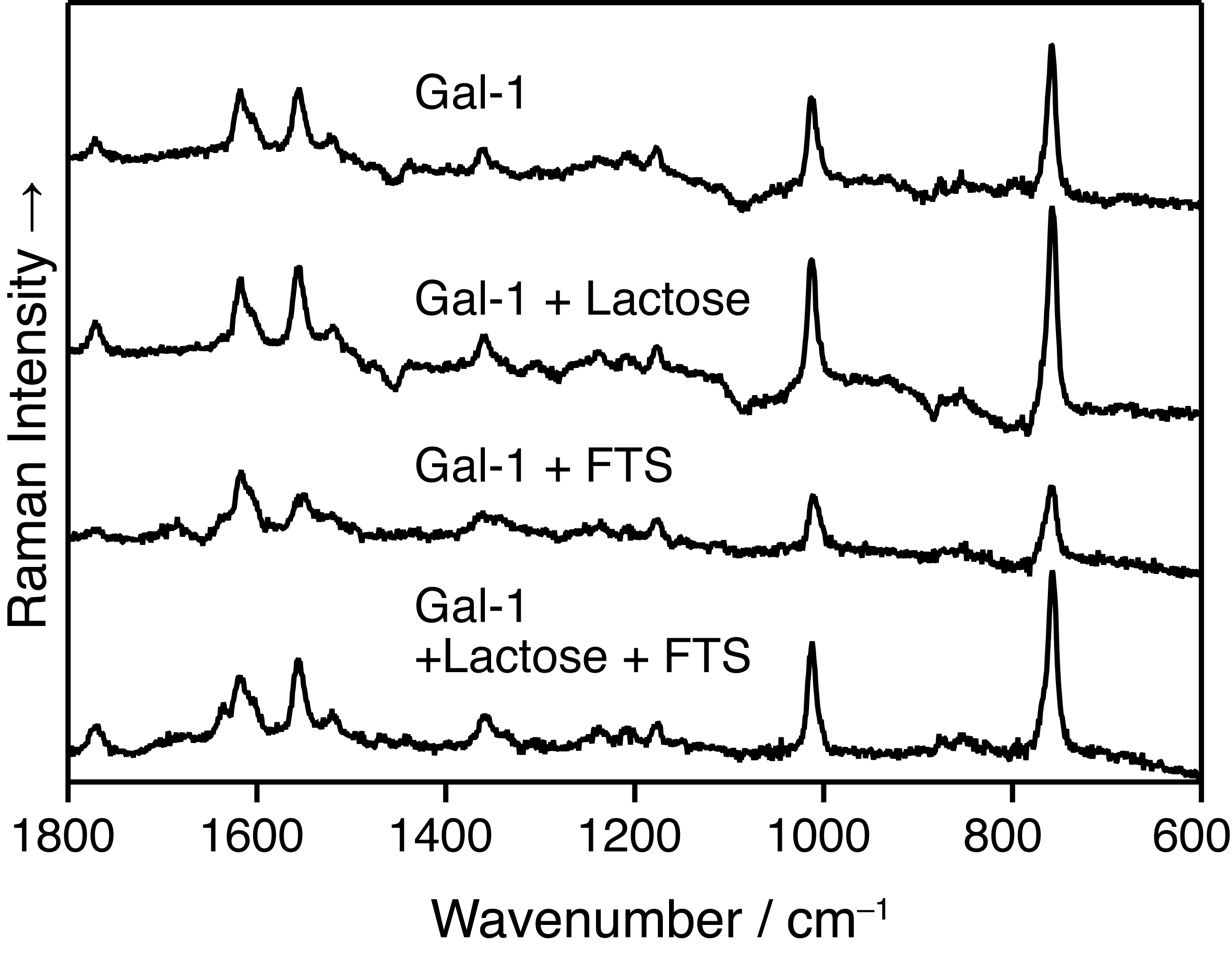


**Figure S2** Raman spectra measured with 229-nm excitation beam of Gal-1 (top), Gal-1 + lactose (second row), Gal-1 + FTS (third row), and Gal-1 + lactose + FTS (bottom).


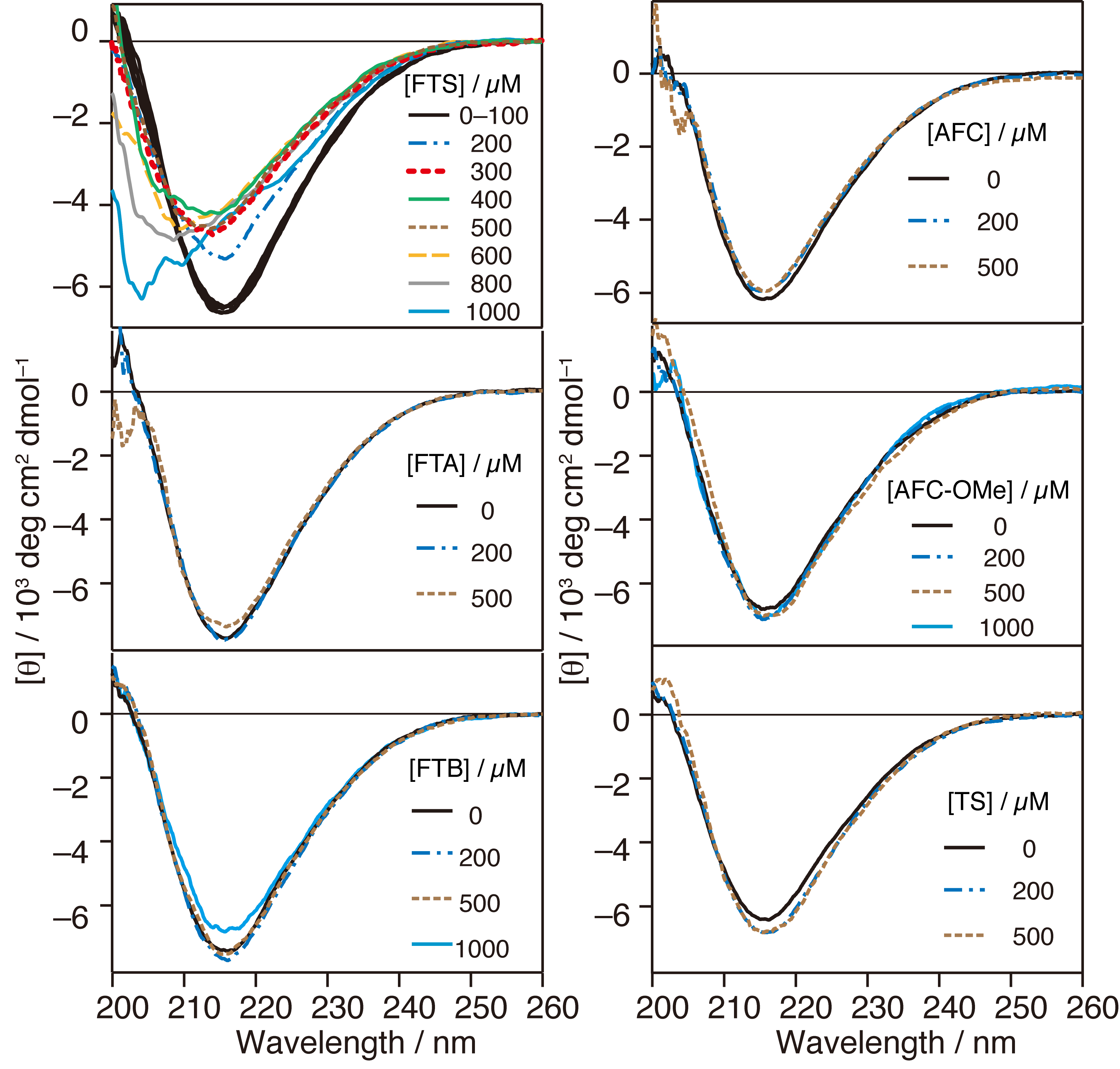


**Figure S3** CD spectra of Gal-1 at different concentrations of FTS (left, top), FTA (left, middle), FTB (left, bottom), AFC (right, top), AFC-OMe (right, middle), and TS (right, bottom).

REFERENCES

1 Marciano, D. *et al.* Farnesyl derivatives of rigid carboxylic acids –Inhibitors of ras-dependent cell growth. *J. Med. Chem.* 38, 1267-1272 (1995).

2 Van Der Spoel, D. *et al.* GROMACS: fast, flexible, and free. *J. Comput. Chem.* 26, 1701-1718 (2005).

3 Malde, A. K. *et al.* An automated force field topology builder (ATB) and repository: version 1.0. *J. Chem. Theory Comput.* 7, 4026-4037 (2011).

4 Koziara, K. B., Stroet, M., Malde, A. K. & Mark, A. E. Testing and validation of the automated topology builder (ATB) version 2.0: prediction of hydration free enthalpies. *J. Comput. Aided Mol. Des.* 28, 221-233 (2014).

5 McNicholas, S., Potterton, E., Wilson, K. S. & Noble, M. E. Presenting your structures: the CCP4mg molecular-graphics software. *Acta Crystallogr. sect. D* D67, 386-394 (2011).

6 Nesmelova, I. V. *et al.* Lactose binding to galectin-1 modulates structural dynamics, increases conformational entropy, and occurs with apparent negative cooperativity. *J. Mol. Biol.* 397, 1209-1230 (2010).

7 Darden, T., York, D. & Pedersen, L. Particle mesh Evald: an N•log(N) method for Evald sums in large systems. *J. Chem. Phys.* 98, 10089-10092 (1993).

8 Hess, B., Bekker, H., Berendsen, H. J. C. & Fraaije, J. G. E. M. LINCS: a linear constraint solver for molecular simulations. *J. Comput. Chem.* 18, 1463-1472 (1997).

9 Yu, X. *et al.* Redox state influence on human galectin-1 function. *Biochimie* 116, 8-16 (2015).

10 Matsuno, M. & Takeuchi, H. Effects of hydrogen bonding and hydrophobic interactions on the ultraviolet resonance Raman intensities of indole ring vibrations. *Bull. Chem. Soc. Jpn* 71, 851-857 (1998).

11 Di Lella, S. *et al.* Critical role of the solvent environment in galectin-1 binding to the disaccharide lactose. *Biochemistry* 48, 786-791 (2009).

12 Lopez-Lucendo, M. F. *et al.* Growth-regulatory human galectin-1: crystallographic characterisation of the structural changes induced by single-site mutations and their impact on the thermodynamics of ligand binding. *J. Mol. Biol.* 343, 957-970 (2004).
